# Adding Estimated Cardiorespiratory Fitness to the Framingham Risk Score and Mortality Risk in a Korean Population-Based Cohort Study

**DOI:** 10.3390/ijerph19010510

**Published:** 2022-01-03

**Authors:** Inhwan Lee, Jeonghyeon Kim, Hyunsik Kang

**Affiliations:** College of Sport Science, Sungkyunkwan University, Suwon 16419, Korea; ansh00@naver.com (I.L.); zzagkim115@naver.com (J.K.)

**Keywords:** cardiovascular disease, cardiorespiratory fitness, mortality, Framingham risk score, Koreans

## Abstract

Background: The added value of non-exercise-based estimation of cardiorespiratory fitness (eCRF) to cardiovascular disease (CVD) risk factors for mortality risk has not been examined in Korean populations. Methods: This population-based prospective cohort study examined the relationship of the 10-year Framingham risk score (FRS) for CVD risk and eCRF with all-cause and CVD mortality in a representative sample of Korean adults aged 30 years and older. Data regarding a total of 38,350 participants (16,505 men/21,845 women) were obtained from the 2007–2015 Korea National Health and Nutrition Examination Survey (KNHANES). All-cause and CVD mortality were the main outcomes. The 10-year FRS point sum and eCRF level were the main exposures. Results: All-cause and CVD mortality was positively correlated with the 10-year FRS point summation and inversely correlated with eCRF level in this study population. The protective of high eCRF against all-cause and CVD mortality was more prominent in the middle and high FRS category than in the low FRS category. Notably, the FRS plus eCRF model has better predictor power for estimating mortality risk compared to the FRS only model. Conclusions: The current findings indicate that eCRF can be used as an alternative to objectively measured CRF for mortality risk prediction.

## 1. Introduction

Cardiovascular disease (CVD) is the leading cause of global death, especially in low- and middle-income countries [1]. Traditional risk factors, such as hypertension, diabetes, cigarette smoking, family history of premature CVD, chronic kidney disease, and obesity, are well-established predictors of morbidity and mortality in Western [2,3] and Asian populations [4,5]. Thus, identifying individuals at increased CVD risk as early as possible is critical so that an appropriate therapeutic strategy can be timely implemented.

The Framingham risk score (FRS) for estimating 10-year CVD risk was developed with the data of coronary risk factors obtained from the Framingham Heart Study [6]. The 10-year FRS is the most widely used and validated tool for estimating CVD risk score [7,8]. However, the 10-year FRS does not account for physical activity (PA) and cardiorespiratory fitness (CRF) although they are well-established predictors for morbidity and mortality [9,10].

CRF is defined as the oxygen-delivering capacity of the circulatory and respiratory systems into skeletal muscles during sustained physical activity. Substantial evidence supporting CRF as an independent determinant of CVD mortality exists in the literature [11,12]. For example, a 1-unit increase in metabolic equivalent of task (MET; approximating 3.5 mL O_2_/kg/min) at baseline was associated with an 18% decrease in 30-year CVD mortality in FRS-based low-risk adults [2]. Higher CRF was significantly associated with lower CVD mortality risk in a 10-year follow-up study [13]. The inverse relationship between CRF and CVD mortality has been investigated systematically and well-summarized in meta-analysis studies [14,15].

In South Korea, CVD is the leading cause of mortality second to malignancy. The predictive role of CVD risk factors for estimating mortality risk has been reported [15,16]. However, only a handful number of population-based studies has examined the potential of CRF as a predictor for all-cause and CVD mortality [17,18]. Furthermore, the addition of estimated CRF (eCRF) to the 10-year FRS for predicting mortality risk in Korean populations is not known.

Together, poor CRF along with the traditional risk factors is now considered as an important indicator of mortality risk in Korean adults. To the best of our knowledge, however, the added value of eCRF to the 10-year FRS for estimating mortality risk in South Korea has not been examined in previous studies. Therefore, in this population-based cohort study, the prognostic value of adding eCRF to the 10-year FRS was investigated by examining improvement in prediction of all-cause and CVD mortality risk.

## 2. Materials and Methods

### 2.1. Study Population and Database Information

The Korea National Health and Nutrition Examination Survey (KNHANES) is a nationwide surveillance system designed to assess health and nutritional status of Koreans in South Korea (http://knhanes.cdc.go.kr/ accessed on 5 May 2021). The detailed design and procedures of the KNHANES are described elsewhere [19]. In brief, the survey includes three parts: health examination, health interview, and nutrition survey. With consent obtained from participants (response rate of 92.1%), the KNHANES 2007–2015 data were linked to death certificates and medical records from 1 January 2007 to 31 December 2016. All participants provided informed consent and the survey was approved by the Ethics Committee of the Korea Centre for Disease Control and Prevention (2007-02CON-04-P, 2008-04EXP-01-C, 2009-01COM-03-2C, 2010-02CON-21-C, 2011-02CON-06-C, 2012-01EXP-01-2C, 2013-07CON-03-4C, and 2013-12EXP-03-5C).

**Figure 1:**
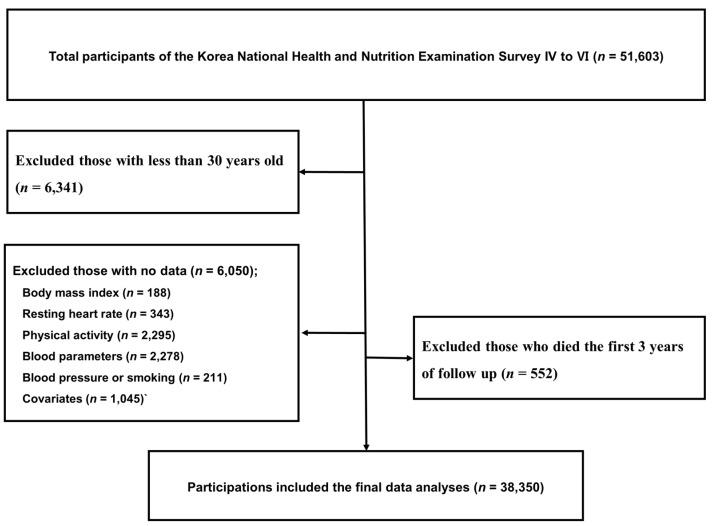
A flow chart of selection of study participants.

The current study included KNHANES participants who responded to the survey from 2007 to 2015, and consented to mortality follow-up (*n* = 51,603). Among the participants, those who were aged <30 years (*n* = 6341) were excluded because no parameters were available to calculate the FRS. In addition, subjects with missing or no data for BMI (*n* = 188), resting HR (*n* = 343), physical activity (*n* = 2295), blood chemistry (*n* = 2278), blood pressure or smoking (*n* = 211) or covariates (i.e., income, education, marriage, drinking, menopause status, chronic diseases, and hospitalization) (*n* = 1045) were excluded. Lastly, subjects who died during the first 3 years of follow-up (*n* = 552) were excluded to minimize the influence of preexisting health conditions on mortality risk. Consequently, a total of 38,350 participants (16,505 men/21,845 women) were included in the final data analysis (Figure 1).

### 2.2. Determination of Anthropometrics and CVD Risk Factors

Height and weight were measured using an automatic height scale (SECA-225, SECA, Hamburg, Germany) and a weight scale (GL-6000-20, G tech, Uijeongbu, Korea), respectively, and body mass index (BMI) was calculated by dividing weight (kg) by height (m^2^). Resting heart rate (RHR) was obtained by counting pulse rate at the right radial for 15 s and multiplying it by 4. Physical activity was assessed using the Korean version of the international physical activity questionnaire short form [20].

For blood chemistry, 13 mL of venous blood was collected from the median cubital vein of the non-dominant arm after an 8 h fast. Blood concentrations of high-density lipoprotein cholesterol (HDLC), low-density lipoprotein cholesterol (LDLC), total cholesterol (TC), fasting blood glucose (FBG) were assessed using standard procedures [19]. Concentrations of LDLC was calculated using the Friedewald formula [21]; LDLC (mg/dL) = TC-HDLC-TG/5. Blood pressure was measured 3 times at the right brachial artery with a mercury sphygmomanometer (Baumanometer desk model 0320, Baum, NY, USA) in a comfortable sitting position. The average of 2–3 measurements was recorded. Use of antihypertensive therapy, current/past smoking, and presence of diabetes was assessed using a health questionnaire. Smoking was defined as currently smoking or having had smoked more than 100 cigarettes in the past. Diabetes was defined as fasting blood glucose of ≥126 mg/dL or physician-diagnosed diabetes.

### 2.3. Determination of the 10-Year FRS and eCRF

The 10-year FRS was calculated based on sex, age, TC, HDLC, SBP, use of antihypertensive therapy, smoking, and presence of diabetes, as described previously [22]. The FRS point summation was classified as low (<10%), moderate (10–20%), or high (>20%) risk.

eCRF was determined using the algorithm developed by Jurca et al. [23]; eCRF (METs) = (ex (women = 0, men = 1) × 2.77) − (age (years) × 0.10) − (BMI (kg/m^2^) × 0.17) − (RHR (beats/min) × 0.03) + (physical activity score × 1.00) + 18.07. Then, receiver operating characteristic (ROC) analysis was conducted to evaluate the gender-specific relationship between individual eCRF values and all-cause mortality. Area under the curve (AUC) value of eCRF for predicting all-cause mortality was 0.754 (95% CI = 0.737–0.770, *p* < 0.001) in males and 0.776 (95% CI = 0.757–0.796, *p* < 0.001) in females. The optimal cut-off value for all-cause mortality was 9.16 and 6.14 METs for men and women, respectively. The sensitivity and specificity of the cut-off values were 0.587 and 0.786, respectively, for men, and 0.668 and 0.776, respectively, for women (Supplemental Tables S1 and S2).

### 2.4. Determination of Mortality

Main outcomes were all-cause and CVD mortality, which were defined as death from all causes or CVD. Date of causes of death from 1 October 2007 to 31 December 2018 were identified by medical records filed at the Korean Statistical Information Service (KOSIS). Using the *International Classification of Disease*, 10th version (ICD-10), all-cause mortality (*n* = 1474) and deaths from cardiovascular diseases (I00-I99) (*n* = 325) were identified. Follow-up time was defined as the period from the baseline visit to the day of death for participants who died or to the last contact date for those who did not experience the outcome event (censored).

### 2.5. Determination of Covariates

The covariates used in the study were age, sex, household income, education (i.e., less than elementary, middle school, high school, college and higher), marital status (i.e., married or widowed/divorced or unmarried), residence area (i.e., urban vs. rural). The covariates were assessed at baseline using a questionnaire [19].

### 2.6. Statistics

Characteristics of study participants were compared using analysis of variance and χ^2^ tests and presented as mean ± standard deviation (SD) for continuous variables and number (percentages) for categorical variables. Death rate per 1000 person/years of follow-up was calculated for e-CRF level and the 10-year FRS point sum.

Cox proportional hazards model was used to estimate hazard ratio (HR) and 95% confidence intervals (CIs) for all-cause and CVD mortality according to 10-year FRS category (i.e., low or moderate or high risk) or eCRF level (i.e., unfit vs. fit) in two models. Model 1 was adjusted for age and sex. Model 2 was additionally adjusted for household income, education, marital status, and residence area. The Kaplan–Meier procedure with log-rank tests was used to estimate mortality functions according to number of baseline lifestyle risk factors. Survival time was measured as the time from the baseline survey to death or the censor point (31 December 2018). In addition, the significance of multiplicate interaction between eCRF and FRS levels for all-cause and CVD mortality was tested by adding cross-products term in the Cox proportional hazard models.

The ROC curves were plotted with the MedCalc statistical software (version 20.009, MedCalc Software Ltd., Ostend, Belgium) to determine if significant improvement in the predictive accuracy of mortality existed when adding eCRF to the FRS point sum. The χ^2^ test was used to determine significant difference in AUC between the FRS plus eCRF model, and the FRS only model for estimating mortality risk.

## 3. Results

As shown in Table 1, there were 5.26 deaths per 1000 person/years (PY) in total, 7.27 deaths per 1000 PY in men, and 3.75 deaths per 1000 PY in women. In general, males were older (*p* = 0.027), heavier (*p* < 0.001), had higher income (*p* < 0.001), higher education level (*p* < 0.001), tended to be married (*p* < 0.001) and religious (*p* < 0.001) compared with females. Males were more active (*p* < 0.001), had higher SBP (*p* < 0.001), higher smoking rate (*p* < 0.001), higher at-risk alcohol consumption (*p* < 0.001), and higher rates of diabetes (*p* < 0.001) with lower resting heart rate (*p* < 0.001), lower HDLC (*p* < 0.001), lower LDLC (*p* < 0.001), and lower TC (*p* < 0.001) than females.

Table 2 shows the descriptive statistics of study participants dichotomized based on eCRF. Fit individuals were younger (*p* < 0.001), had higher income (*p* < 0.001) and higher education level (*p* < 0.001), tended to be married (*p* < 0.001), reside in urban areas (*p* < 0.001), had lower BMI (*p* < 0.001), lower LDLC (*p* < 0.001), lower TC (*p* < 0.001), lower SBP (*p* < 0.001), lower smoking rate (*p* < 0.001), and lower rate of diabetes (*p* < 0.001) with higher HDLC (*p* < 0.001) than did unfit individuals.

**Figure 2:**
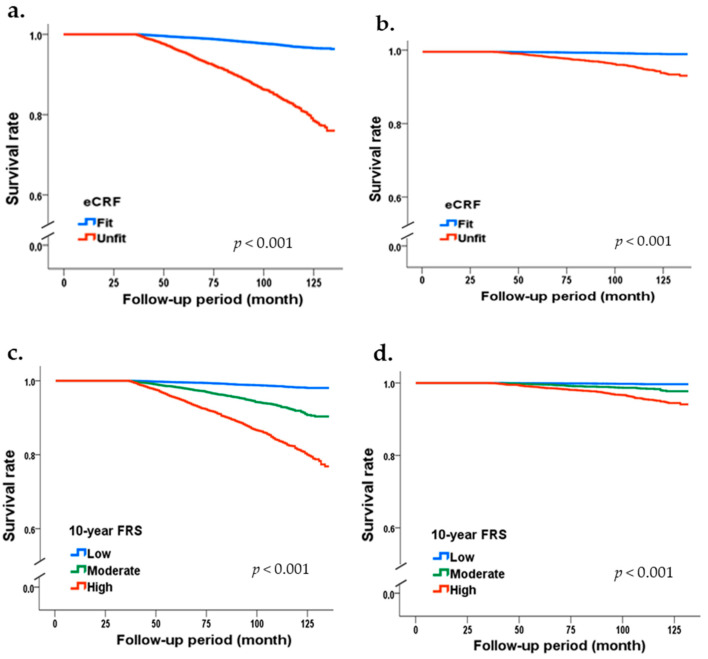
The Kaplan–Meier survival curves for all-cause and cardiovascular disease (CVD) mortality by estimated cardiorespiratory fitness (eCRF) and 10-yr Framingham risk score (FRS): (**a**) eCRF and all-cause mortality; (**b**) eCRF and CVD mortality; (**c**) FRS and all-cause mortality, and (**d**) FRS and CVD mortality.

Table 3 shows the mortality risk stratified based on eCRF and the FRS. Fit individuals had lower all-cause (HR (95% CI) = 0.74 (0.66–0.84), *p* < 0.001) and CVD mortality (HR (95% CI) = 0.77 (0.68–0.87, *p* < 0.001) compared with unfit individuals (HR = 1). The lower HR remained significant for all-cause (HR (95% CI) = 0.62 (0.47–0.80), *p* < 0.001) and CVD mortality (HR (95% CI) = 0.64 (0.49–0.83, *p* = 0.001) even after adjustments for all covariates. In addition, based on FRS category, a positive linear trend was observed for all-cause (HR (95% CI) = 1.14 (0.95–1.36) and HR (95% CI) = 1.31 (1.07–1.60), *p* = 0.025), and CVD mortality (HR (95% CI) = 1.31 (0.87–1.96) and HR (95% CI) = 2.16 (1.41–3.30), respectively, *p* < 0.001). Furthermore, the linear trend in FRS category remained significant for all-cause (HR (95% CI) = 1.08 (0.91–1.30), HR (95% CI) = 1.26 (1.03–1.54, respectively, *p* = 0.046), and CVD mortality (HR (95% CI) = 1.25 (0.83–1.87) and HR (95% CI) = 2.07 (1.36–3.16, respectively, *p* < 0.001) even after adjustments for all covariates. As illustrated in Figure 2, the Kaplan–Meier mortality functions showed that the survival rates of all-cause and CVD mortality decreased significantly by decremental eCRF (from fit to unfit) and incremental FRS (from low to high), respectively.

Table 4 represents HRs and 95% CI for all-cause and CVD mortality by eCRF and FRS. There were significant interaction effects between eCRF and FRS on all-cause and CVD mortality in all models (*p* < 0.001 and *p* < 0.001, respectively). With respect to all-cause mortality, the negative impact of elevated FRS was found to be significant in unfit category but not in fit category. Compared to the combination of fit eCRF and low FRS (HR = 1), the combination of unfit eCRF and moderate FRS and the combination of unfit eCRF and high FRS were associated with the higher risk of all-cause mortality (HR = 1.21, *p* < 0.001 and HR = 1.34, *p* < 0.001, respectively) in the fully adjusted model. With respect to CVD mortality, the negative impact of elevated FRS was observed in the combination of fit eCRF and high FRS (HR = 1.53, *p* < 0.001) as well as the combination of unfit eCRF and moderate FRS (HR = 1.55, *p* < 0.001) and the combination of unfit eCRF and high FRS (HR = 2.55, *p* < 0.001).

**Figure 3:**
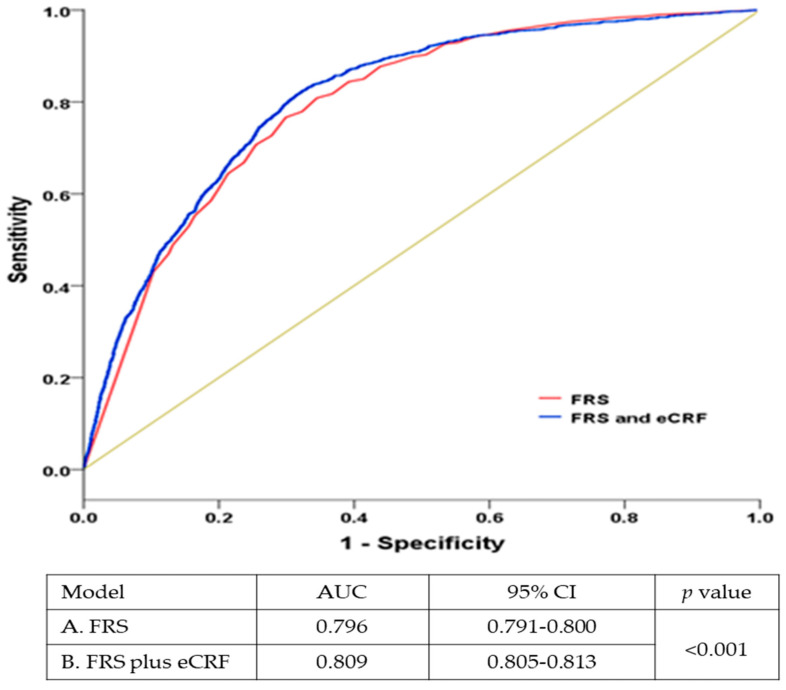
Receiver operating characteristic curve (AUC) comparing the predictive ability of the Framingham risk score (FRS) (Model A) compared to the FRS plus estimated cardiorespiratory fitness (eCRF) (Model B).

Figure 3 shows the ROC curves plotted for the FRS only model and the FRS plus eCRF model. The AUC for the FRS plus e-CRF model was significantly greater (*p* < 0.001) than that for the FRS only model, indicating that the FRS plus eCRF model compared with the FRS only model has higher predictive power for estimating all-cause and CVD mortality.

## 4. Discussion

In this population-based prospective cohort study, we found that all-cause and CVD mortality were positively correlated with the 10-year FRS and inversely correlated with eCRF in Korean adults. In addition, we found that the protective effect of high eCRF against all-cause and CVD mortality was more prominent in the moderate and high FRS category than in the low FRS category, suggesting that fitness promotion via regular exercise may protect the premature death risks from elevated FRS. Notably, the FRS plus eCRF model has better predictive power for all-cause and CVD mortality compared with the FRS only model, implying the added value of eCRF to the traditional risk factor model.

In agreement with the current findings of the study, CRF has been well-established as an independent predictor of CVD mortality in healthy adults [14] as well as in CVD patients [24]. Specifically, Imboden et al. [25] showed that a 1-unit increase in MET was associated with 20% and 38% decreases in 30-year mortality risk for males and females, respectively. Cao et al. [12] showed that CRF was associated inversely with all-cause and cancer mortality in a US Baby Boomers and Generation Xers-based cohort study. The association between higher CRF and lower mortality also is observed in in a 10-year prospective cohort study involving 59,941 Koreans 30–84 years [17], and in a retrospective cohort study of 18,775 Korean men [18].

Although CRF is a well-known predictor of all-cause and CVD mortality risk, it is not routinely checked during clinical visits because specialized equipment, trained personnel, and sufficient time are needed. However, in recent studies, CRF was estimated with an acceptable accuracy using easily obtained health indicators [23], and it has been used as a parameter for predicting morbidity and mortality risk. In a population-based cohort study involving 12,834 participants aged 20 to 86 years, Zhang et al. [26] showed that higher eCRF was significantly associated with lower all-cause and CVD mortality risk in males and females. Wang et al. [27] also showed that a 1-MET increase in eCRF was associated with 30% and 27% lower risk of all-cancer mortality in males and females, respectively. In a prospective cohort study involving 29,850 men from the Aerobics Center Longitudinal Study, Gander et al. [28] showed that higher eCRF was associated with lower coronary heart disease (CHD) and lower CHD mortality. In our previous study with Korean older adults, we also found that higher eCRF was significantly associated with lower all-cause mortality risk [29]. However, we are not sure if eCRF can replace direct measurement of CRF as a predictor for mortality risk, which should be further investigated in a future study involving both predictors.

The prognostic value of adding eCRF to the FRS only model observed in the current study is of particular interest and supports the findings from previous studies. For example, Gupta et al. [24] examined the effect of adding CRF to traditional risk factors on risk classification in 66,371 healthy adults and found that the addition of CRF to the traditional risk factors improved classification of short- and long-term risk for CVD mortality. In a cross-sectional study involving 6962 patients, Myers et al. [30] showed that addition of physical activity and CRF to the traditional risk factor model improved risk classification accuracy for mortality by 22.8% and 43.5%, respectively. The prognostic role of adding other health indicators to the CVD risk factors-based model for mortality risk has been reported in previous studies [31,32]. Likewise, the role of adding other health indicators to the CVD risk factors-based model for predicting mortality risk has also been reported in previous studies [31,32]. For example, the algorithm for eCRF used in the current study includes age, sex, PA, RHR, and BMI as parameters. Among them, PA, RHR, and BMI are additional predictors that are not included in the FRS model. Therefore, the added value of eCRF to the FRS only model may imply the potential of the three parameters. In support of this notion, elevated RHR is a significant predictor of all-cause mortality in ambulatory patients with heart failure [33] and middle-aged populations [34,35]. In addition, mortality risk is significantly and independently associated with overweight/obesity [36,37], and physical inactivity [9,10] in different populations. Together, the findings from the current and previous studies suggest that adding eCRF to the traditional risk factor-based models may positively contribute to the accuracy for predicting mortality risk.

Several explanations are possible for the protective effect of high CRF against increased all-cause and CVD mortality risk associated with the traditional risk factors. CVD risk factors either individually and/or additively contribute to increased mortality risk via (i) decreased insulin sensitivity and increased insulin resistance, (ii) unfavorable blood lipids and lipoproteins, (iii) obesity/overweight, (iv) inflammatory responses, (v) elevated blood pressure, (vi) increased sympathetic and decreased parasympathetic tone, and (vii) metabolic disorders [38,39,40]. Conversely, obtaining and/or maintaining high CRF through regular physical activity provides morbidity and mortality benefits via improved insulin sensitivity and decreased insulin resistance [41], favorable changes in blood lipids and lipoproteins [42], anti-inflammation [43], decreased blood pressure [44], increased parasympathetic/decreased sympathetic tone [45], and decreased clustering of metabolic risk factors [46].

This study has limitations. First, the cross-sectional nature of the study limits any cause-and-effect explanation for the relationships between exposures and mortality. Second, parameters used to obtain eCRF would change during follow-up. However, only baseline eCRF was used in this study, which could result in an underestimation of the study’s associations. Third, the possibility cannot be excluded that other biomarkers or covariates not included in the current study may mediate or modulate the relationships between exposures and mortality [47]. This study also has strengths. First, this study included a large and representative sample of Korean adults for generalization of study outcomes. Second, the mortality data were gathered from a reliable register. Third, eCRF classification (unfit vs. fit) was based on the ROC analysis of the current data, which would provide a more reliable and reproducible association between the exposure and mortality.

## 5. Conclusions

In summary, we examined the relationship of the 10-year FRS point summation and eCRF levels with all-cause and CVD mortality in a representative sample of Korean adults and found that all-cause and CVD mortality risk was positively correlated with the FRS but inversely correlated with eCRF. The protective effect of high eCRF against all-cause and CVD mortality was noticeable in moderate and high FRS categories. In addition, we found that the FRS plus eCRF model has better predictor power for mortality risk than the FRS only model. Together, the current findings indicate that CRF promotion via physical activity should be encouraged for subjects at increased CVD risk, and incorporating eCRF into the traditional risk factors could be an alternative to objectively measuring CRF for mortality risk prediction.

## Figures and Tables

**Table 1:** Characteristics of study participants.

		Total (*n* = 38,350)	Men (*n* = 16,505)	Women (*n* = 21,845)	*p*-Value
Age (years)		52.5 ± 13.7	52.7 ± 13.6	52.4 ± 13.8	0.027
BMI (kg/m^2^)		23.9 ± 3.3	24.2 ± 3.1	23.7 ± 3.4	<0.001
Income (10,000/won)		329.2 ± 271.2	338.2 ± 270.4	322.3 ± 271.7	<0.001
Educational background, *n* (%)					<0.001
	Elementary or less	10,460 (27.3)	3155 (19.1)	7305 (33.4)	
	Middle	4670 (12.2)	2132 (12.9)	2538 (11.6)	
	High	12,237 (31.9)	5407 (32.8)	6830 (31.3)	
	College or higher	10,983 (28.6)	5811 (35.2)	5172 (23.7)	
Marital status, *n* (%)					<0.001
	Married	31,121 (81.1)	14,405 (87.3)	16,716 (76.5)	
	Widowed/divorced	5383 (14.0)	971 (5.9)	4412 (20.2)	
	Unmarried	1846 (4.9)	1129 (6.8)	717 (3.3)	
Residence area, *n* (%)					<0.001
	Urban	29,550 (77.1)	12,573 (76.2)	16,977 (77.7)	
	Rural	8800 (22.9)	3932 (23.8)	4868 (22.3)	
CVD risk factors					
	HDL-C (mg/dL)	49.1 ± 11.7	46.1 ± 10.9	51.4 ± 11.8	<0.001
	LDL-C (mg/dL)	114.1 ± 33.6	110.4 ± 3.9	117.0 ± 32.3	<0.001
	TC (mg/dL)	190.9 ± 35.9	189.0 ± 35.4	192.4 ± 36.3	<0.001
	SBP (mmHg)	119.8 ± 17.3	122.2 ± 16.0	118.1 ± 18.1	<0.001
	SBP treat, *n* (%)	8209 (21.4)	3486 (21.1)	4723 (21.6)	0.238
	Smoking, *n* (%)	15,004 (39.1)	13,251 (80.3)	1753 (8.0)	<0.001
	Diabetes, *n* (%)	2885 (7.5)	1530 (9.3)	1352 (6.2)	<0.001
Resting heart rate (beats/min)		69.3 ± 9.7	68.5 ± 10.1	69.9 ± 9.3	<0.001
Physically inactive, *n* (%)		25,146 (65.6)	9741 (59.0)	15,405 (70.5)	<0.001
Follow-up time (years)		7.3 ± 2.4	7.3 ± 2.4	7.3 ± 2.4	0.241
Death, n		1474	875	599	<0.001
Person/years (PY)		280,315	120,368	159,946	<0.001
Death rate per 1000 PY		5.3	7.3	3.8	<0.001

BMI: body mass index, HDL-C: high density lipoprotein cholesterol, LDL-C: low density lipoprotein cholesterol, TC: total cholesterol, SBP: systolic blood pressure.

**Table 2:** Demographics of participants stratified by estimated cardiorespiratory fitness (eCRF).

		eCRF		*p*-Value
		Fit (*n* = 29,339)	Unfit *n* = 9011)	
eCRF (METs)		10.1 ± 2.1	6.4 ± 1.7	<0.001
Women, *n* (%)		16,692 (56.9)	5153 (57.2)	0.624
Age (years)		47.7 ± 11.4	68.1 ± 7.6	<0.001
BMI (kg/m^2^)		23.6 ± 3.1	25.0 ± 3.4	<0.001
Income (10,000/won)		367.6 ± 271.2	203.9 ± 230.4	<0.001
Education, *n* (%)				<0.001
	Elementary or less	4861 (16.6)	5599 (62.1)	
	Middle	3394 (11.6)	1276 (14.2)	
	High	10,771 (36.7)	1466 (16.3)	
	College or higher	10,313 (35.1)	670 (7.4)	
Marital status, *n* (%)				<0.001
	Married	24,841 (84.7)	6280 (69.7)	
	Widowed/divorced	2718 (9.3)	2665 (29.6)	
	Unmarried	1780 (6.0)	66 (0.7)	
Residence area, *n* (%)				<0.001
	Urban	23,331 (79.5)	6219 (69.0)	
	Rural	6008 (20.5)	2792 (31.0)	
CVD risk factors				
	HDL-C (mg/dL)	49.9 ± 11.8	46.6 ± 11.1	<0.001
	LDL-C (mg/dL)	113.8 ± 33.0	115.3 ± 35.6	<0.001
	TC (mg/dL)	190.6 ± 35.2	192.0 ± 38.4	0.001
	SBP (mmHg)	117.1 ± 16.4	128.9 ± 17.2	<0.001
	SBP treat, *n* (%)	3940 (13.4)	4269 (47.4)	<0.001
	Smoking, *n* (%)	11,477 (39.1)	3527 (39.1)	0.970
	Diabetes, *n* (%)	1611 (5.5)	1274 (14.1)	<0.001
10-year FRS (%)		8.3 ± 8.7	19.4 ± 9.4	<0.001

eCRF: estimated cardiorespiratory fitness, MET: metabolic equivalent task, BMI: body mass index, CVD: cardiovascular disease, FRS: Framingham risk score, HDL-C: high density lipoprotein cholesterol, LDL-C: low density lipoprotein cholesterol, TC: total cholesterol, SBP: systolic blood pressure.

**Table 3:** All-cause and CVD mortality risks stratified by 10-year Framingham risk score (FRS) and estimated cardiorespiratory fitness (eCRF).

		N	Number of Deaths	Death Rate ^a^	HR (95% CI) for All-Cause Mortality ^b^	HR (95% CI) for All-Cause Mortality ^c^	HR (95% CI) for CVD Mortality ^b^	HR (95% CI) for CVD Mortality ^c^
eCRF								
	Fit	29,339	562	2.6	1	1	1	1
	Unfit	9011	912	15.1	1.35 (1.20–1.52)	1.31 (1.16–1.47)	1.62 (1.24–2.12)	1.57 (1.20–2.05)
*p*-value					<0.001	<0.001	<0.001	0.001
10-year FRS								
	Low	22,653	230	1.4	1	1	1	1
	Moderate	7443	344	6.4	1.14 (0.95–1.36)	1.08 (0.91–1.30)	1.31 (0.87–1.96)	1.25 (0.83–1.87)
	High	8254	900	15.5	1.31 (1.07–1.60)	1.26 (1.03–1.54)	2.16 (1.41–3.30)	2.07 (1.36–3.16)
*p*-value for trend					0.025	0.046	<0.001	<0.001

^a^ Deaths per 1000 person/years of follow-up. ^b^ Adjusted for age and sex. ^c^ Adjusted for household income, marital status, and residence area. HR: hazard ratio; CI: confidence interval. Low: <10% of 10-year FRS; moderate: 10–20% of 10-year FRS; high: ≥20% of 10-year FRS.

**Table 4:** The combined associations of estimated cardiorespiratory fitness (eCRF) and 10-year Framingham risk score (FRS) with all-cause and cardiovascular disease (CVD) mortality.

				HR (95% CI) for All-Cause Mortality		HR (95% CI) for CVD Mortality	
	Total	Number of Deaths	Death Rate ^a^	Model 1	Model 2	Model 1	Model 2
Fit							
Low FRS	20,635	169	1.1	1 (ref)	1 (ref)	1 (ref)	1 (ref)
Moderate FRS	4586	126	3.6	0.89 (0.67–1.14)	0.84 (0.66–1.07)	1.05 (0.58–1.90)	0.98 (0.54–1.77)
High FRS	3556	267	9.4	1.03 (0.80–1.31)	0.98 (0.77–1.26)	1.62 (0.92–2.86)	1.53 (0.87–2.69)
Unfit							
Low FRS	1788	61	4.9	0.84 (0.61–1.15)	0.79 (0.58–1.08)	1.07 (0.54–2.09)	0.98 (0.50–1.93)
Moderate FRS	2513	218	11.9	1.32 (1.04–1.67)	1.21 (0.95–1.54)	1.70 (0.98–2.93)	1.55 (0.90–2.67)
High FRS	3798	633	21.4	1.44 (1.13–1.84)	1.34 (1.05–1.71)	2.73 (1.58–4.72)	2.50 (1.45–4.31)
P for interaction				<0.001	<0.001	<0.001	<0.001

^a^ Deaths per 1000 person/years of follow-up. Model 1 adjusted for age and sex. Model 2 adjusted for household income, marital status, and residence area. HR: hazard ratio; CI: confidence interval. Low: <10% of 10-year FRS; moderate: 10~20% of 10-year FRS; high: >20% of 10-year FRS.

## Data Availability

The datasets used and/or analyzed during the current study are available from the corresponding author on request.

## Supplementary Materials

The following are available online at https://www.mdpi.com/article/10.3390/ijerph19010510/s1, Table S1: The receiver operating curve analysis of estimated cardiorespiratory fitness for all-cause mortality; Table S2: Cut-off values of estimated cardiorespiratory fitness for all-cause mortality.

## References

[1] World Health Organization (2021). Fact Sheets; Cardiovascular Diseases (CVDs). https://www.who.int/news-room/fact-sheets/detail/cardiovascular-diseases-(cvds).

[2] Barlow C.E., Defina L.F., Radford N.B., Berry J.D., Cooper K.H., Haskell W.L., Jones L.W., Lakoski S.G. (2021). Cardiorespiratory fitness and long-term survival in “low-risk” adults. J. Am. Heart Assoc..

[3] Rawshani A., Rawshani A., Rawshani A., Franzén S., Sattar N., Eliasson B., Svensson A.M., Zethelius B., Miftaraj M., McGuire D.K. (2018). Risk factors, mortality, and cardiovascular outcomes in patients with type 2 diabetes. N. Engl. J. Med..

[4] Kim S.M., Kim S.M., Lee G., Choi S., Kim K., Jeong S.M., Son J.S., Yun J.M., Kim S.G., Hwang S.S. (2020). Association of early-onset diabetes, prediabetes and early glycaemic recovery with the risk of all-cause and cardiovascular mortality. Diabetologia.

[5] Li T.C., Li C.I., Liu C.S., Lin W.Y., Lin C.H., Yang S.Y., Lin C.C. (2020). Derivation and validation of 10-year all-cause and cardiovascular disease mortality prediction model for middle-aged and elderly community-dwelling adults in Taiwan. PLoS ONE.

[6] Wilson P.W., D’Agostino R.B., Levy D., Belanger A.M., Silbershatz H., Kannel W.B. (1998). Prediction of coronary heart disease using risk factor categories. Circulation.

[7] Bitton A., Gaziano T. (2020). The Framingham heart study’s impact on global risk assessment. Prog. Cardiovasc. Dis..

[8] Collins G., Altman D. (2010). An independent and external validation of QRISK2 cardiovascular disease risk score: A prospective open cohort study. BMJ.

[9] Äijö M., Kauppinen M., Kujala U.M., Parkatti T. (2016). Physical activity, fitness, and all-cause mortality: An 18-year follow-up among old people. J. Sport Health Sci..

[10] Min C., Yoo D.M., Wee J.H., Lee H.J., Byun S.H., Choi H.G. (2020). Mortality and cause of death in physical activity and insufficient physical activity participants: A longitudinal follow-up study using a national health screening cohort. BMC Public Health.

[11] Imboden M.T., Harber M.P., Whaley M.H., Finch W.H., Bishop D.L., Kaminsky L.A. (2018). Cardiorespiratory fitness and mortality in healthy men and women. J. Am. Coll. Cardiol..

[12] Cao C., Yang L., Cade W.T., Racette S.B., Park Y., Cao Y., Friedenreich C.M., Hamer M., Stamatakis E., Smith L. (2020). Cardiorespiratory fitness is associated with early death among healthy young and middle-aged baby boomers and generation Xers. Am. J. Med..

[13] Mitchell J.A., Bornstein D.B., Sui X., Hooker S.P., Church T.S., Lee C.D., Lee D.C., Blair S.N. (2010). The impact of combined health factors on cardiovascular disease mortality. Am. Heart J..

[14] Ross R., Blair S.N., Arena R., Church T.S., Després J.P., Franklin B.A., Haskell W.L., Kaminsky L.A., Levine B.D., Lavie C.J. (2016). Importance of assessing cardiorespiratory fitness in clinical practice: A case for fitness as a clinical vital sign: A scientific statement from the American Heart Association. Circulation.

[15] Schmid D., Leitzmann M.F. (2015). Cardiorespiratory fitness as predictor of cancer mortality: A systematic review and meta-analysis. Ann. Oncol..

[16] Kim J.Y., Ko Y.J., Rhee C.W., Park B.J., Kim D.H., Bae J.M., Shin M.H., Lee M.S., Li Z.M., Ahn Y.O. (2013). Cardiovascular health metrics and all-cause and cardiovascular disease mortality among middle-aged men in Korea: The Seoul male cohort study. J. Prev. Med. Public Health.

[17] Yun J.E., Won S., Kimm H., Jee S.H. (2012). Effects of a combined lifestyle score on 10-year mortality in Korean men and women: A prospective cohort study. BMC Public Health.

[18] Park M.S., Chung S.Y., Chang Y., Kim K. (2009). Physical activity and physical fitness as predictors of all-cause mortality in Korean men. J. Korean Med. Sci..

[19] Kweon S., Kim Y., Jang M.J., Kim Y., Kim K., Choi S., Chun C., Khang Y.H., Oh K. (2014). Data resource profile: The Korea National Health and Nutrition Examination Survey (KNHANES). Int. J. Epidemiol..

[20] Oh J.Y., Yang Y.J., Kim B.S., Kang J.H. (2007). Validity and reliability of Korean version of international physical activity questionnaire (IPAQ) short form. J. Korean Acad. Fam. Med..

[21] Friedewald W.T., Levy R.I., Fredrickson D.S. (1972). Estimation of low-density lipoprotein cholesterol in plasma without use of preparative ultracentrifuge. Clin. Chem..

[22] D’Agostino R.B.S., Vasan R.S., Pencina M.J., Wolf P.A., Cobain M., Massaro J.M., Kannel W.B. (2008). General cardiovascular risk profile for use in primary care: The Framingham Heart Study. Circulation.

[23] Jurca R., Jackson A.S., LaMonte M.J., Morrow J.R., Blair S.N., Wareham N.J., Haskell W.L., van Mechelen W., Church T.S., Jakicic J.M. (2005). Assessing cardiorespiratory fitness without performing exercise testing. Am. J. Prev. Med..

[24] Gupta S., Rohatgi A., Ayers C.R., Willis B.L., Haskell W.L., Khera A., Drazner M.H., de Lemos J.A., Berry J.D. (2011). Cardiorespiratory fitness and classification of risk of cardiovascular disease mortality. Circulation.

[25] Imboden M.T., Harber M.P., Whaley M.H., Finch W.H., Bishop D.A., Fleenor B.S., Kaminsky L.A. (2019). The influence of change in cardiorespiratory fitness with short-term exercise training on mortality risk from the Ball State Adults Fitness Longitudinal Lifestyle Study (BALL ST). Mayo Clin. Proc..

[26] Zhang Y., Zhang J., Zhou J., Ernstsen L., Lavie C.J., Hooker S.P., Sui X. (2017). Nonexercise estimated cardiorespiratory fitness and mortality due to all causes and cardiovascular disease: The NHANES III study. Mayo Clin. Proc. Innov. Qual. Outcomes.

[27] Wang Y., Chen S., Zhang J., Zhang Y., Ernstsen L., Lavie C.J., Hooker S.P., Chen Y., Sui X. (2018). Nonexercise estimated cardiorespiratory fitness and all-cancer mortality: The NHANES III Study. Mayo Clin. Proc..

[28] Gander J.C., Sui X., Hébert J.R., Lavie C.J., Hazlett L.J., Cai B., Blair S.N. (2017). Addition of estimated cardiorespiratory fitness to the clinical assessment of 10-year coronary heart disease risk in asymptomatic men. Prev. Med. Rep..

[29] Song M., Lee I., Kang H. (2019). Cardiorespiratory Fitness without Exercise Testing Can Predict All-Cause Mortality Risk in a Representative Sample of Korean Older Adults. Int. J. Environ. Res. Public Health.

[30] Myers J., Nead K.T., Chang P., Abella J., Kokkinos P., Leeper N.J. (2015). Improved reclassification of mortality risk by assessment of physical activity in patients referred for exercise testing. Am. J. Med..

[31] Nishimoto M., Tagawa M., Matsui M., Eriguchi M., Samejima K.I., Iseki K., Iseki C., Asahi K., Yamagata K., Konta T. (2019). A prediction model with lifestyle in addition to previously known risk factors improves its predictive ability for cardiovascular death. Sci. Rep..

[32] Lee W.J., Peng L.N., Chiou S.T., Chen L.K. (2017). Physical health indicators improve prediction of cardiovascular and all-cause mortality among middle-aged and older people: A national population-based study. Sci. Rep..

[33] Lau K., Malik A., Foroutan F., Buchan T.A., Daza J.F., Sekercioglu N., Orchanian-Cheff A., Alba A.C. (2021). Resting heart rate as an important predictor of mortality and morbidity in ambulatory patients with heart failure: A systematic review and meta-analysis. J. Card. Fail..

[34] Seccareccia F., Pannozzo F., Dima F., Minoprio A., Menditto A., Lo Noce C., Giampaoli S., Malattie Cardiovascolari Aterosclerotiche Istituto Superiore di Sanita Project (2001). Heart rate as a predictor of mortality: The MATISS project. Am. J. Public Health.

[35] Jensen M.T., Suadicani P., Hein H.O., Gyntelberg F. (2013). Elevated resting heart rate, physical fitness and all-cause mortality: A 16-year follow-up in the Copenhagen Male Study. Heart.

[36] Di Angelantonio E., Bhupathiraju S.N., Wormser D., Gao P., Kaptoge S., Berrington de Gonzalez A., Cairns B.J., Huxley R., Jackson C.L., Global BMI Mortality Collaboration (2016). Body-mass index and all-cause mortality: Individual-participant-data meta-analysis of 239 prospective studies in four countries. Lancet.

[37] Yang Y., Dugué P.A., Lynch B.M., Hodge A.M., Karahalios A., MacInnis R.J., Milne R.L., Giles G.G., English D.R. (2019). Trajectories of body mass index in adulthood and all-cause and cause-specific mortality in the Melbourne collaborative cohort study. BMJ Open.

[38] Adeva-Andany M.M., Martínez-Rodríguez J., González-Lucán M., Fernández-Fernández C., Castro-Quintela E. (2019). Insulin resistance is a cardiovascular risk factor in humans. Diabetes Metab. Syndr..

[39] Agostinis-Sobrinho C.A., Ruiz J.R., Moreira C., Abreu S., Luís L., Oliveira-Santos J., Mota J., Santos R. (2017). Cardiorespiratory fitness and inflammatory profile on cardiometabolic risk in adolescents from the LabMed Physical Activity Study. Eur. J. Appl. Physiol..

[40] Oliveira C., Silveira E.A., Rosa L., Santos A., Rodrigues A.P., Mendonça C., Silva L., Gentil P., Rebelo A.C. (2020). Risk factors associated with cardiac autonomic modulation in obese individuals. J. Obes..

[41] Haufe S., Engeli S., Budziarek P., Utz W., Schulz-Menger J., Hermsdorf M., Wiesner S., Otto C., Haas V., de Greiff A. (2001). Cardiorespiratory fitness and insulin sensitivity in overweight or obese subjects may be linked through intrahepatic lipid content. Diabetes.

[42] Breneman C.B., Polinski K., Sarzynski M.A., Lavie C.J., Kokkinos P.F., Ahmed A., Sui X. (2016). The impact of cardiorespiratory fitness levels on the risk of developing atherogenic dyslipidemia. Am. J. Med..

[43] González-Gil E.M., Santaliestra-Pasías A.M., Buck C., Gracia-Marco L., Lauriam F., Pala V., Molnar D., Veidebaum T., Iacoviello L., Tornaritis M. (2021). Improving cardiorespiratory fitness protects against inflammation in children: The IDEFICS study. Pediatr. Res..

[44] Agostinis-Sobrinho C., Ruiz J.R., Moreira C., Abreu S., Lopes L., Oliveira-Santos J., Mota J., Santos R. (2018). Cardiorespiratory fitness and blood pressure: A longitudinal analysis. J. Pediatr..

[45] Röhling M., Strom A., Bönhof G.J., Roden M., Ziegler D. (2017). Cardiorespiratory Fitness and Cardiac Autonomic Function in Diabetes. Curr. Diab. Rep..

[46] Wedell-Neergaard A.S., Krogh-Madsen R., Petersen G.L., Hansen Å.M., Pedersen B.K., Lund R., Bruunsgaard H. (2018). Cardiorespiratory fitness and the metabolic syndrome: Roles of inflammation and abdominal obesity. PLoS ONE.

[47] Shigdel R., Dalen H., Sui X., Lavie C.J., Wisløff U., Ernstsen L. (2019). Cardiorespiratory fitness and the risk of first acute myocardial infarction: The HUNT study. J. Am. Heart Assoc..

