# Supplementary material for: Adding Estimated Cardiorespiratory Fitness to the Framingham Risk Score and Mortality Risk in a Korean Population-Based Cohort Study
Source: Int J Environ Res Public Health. 2022 Jan 3;19(1):510. doi: 10.3390/ijerph19010510 (PMC8744979; doi:10.3390/ijerph19010510)
Supplement: Supplementary file 1 [file ijerph-19-00510-s001.zip › ijerph-1519193-supplementary.pdf]

Legends of Supplemental Tables.

.

Table S1. The receiver operating curve analysis of estimated cardiorespiratory fitness for all-cause mortality.

..

Sex.	AUC (95% CI).	SE.	Youden's J.	p value..
Men.	0.754 (0.737-0.770).	0.008.	0.373.	<0.001..
Women.	0.776 (0.757-0.796).	0.010.	0.444.	<0.001..

AUC: Under area curve; CI: Confidence interval; SE: Standard error..

Table S2. Cut-off values of estimated cardiorespiratory fitness for all-cause mortality.

Sex	Cut-off value (METs)	Sensitivity	Specificity	PPV	NPV	Accuracy
Men	9.16	0.587	0.786	58.6	78.6	77.5
Women	6.14	0.668	0.776	66.6	77.6	77.3

PPV; positive predictive value; NPV: Negative predictive value.
